# Crop and forest pest metawebs shift towards increased linkage and suitability overlap under climate change

**DOI:** 10.1038/s42003-020-0962-9

**Published:** 2020-05-11

**Authors:** Marc Grünig, Dominique Mazzi, Pierluigi Calanca, Dirk Nikolaus Karger, Loïc Pellissier

**Affiliations:** 1 Agroscope, RD Plant Protection, Wädenswil, Switzerland; 2 Agroscope, RD Agroecology and Environment, Zurich, Switzerland; 3 ETH, Landscape Ecology, Zurich, Switzerland; 4 Swiss Federal Research Institute WSL, Birmensdorf, Switzerland

**Keywords:** Invasive species, Climate-change ecology, Ecological modelling

## Abstract

Global changes pose both risks and opportunities to agriculture and forestry, and biological forecasts can inform future management strategies. Here, we investigate potential land-use opportunities arising from climate change for these sectors in Europe, and risks associated with the introduction and establishment of novel insect pests. Adopting a metaweb approach including all interaction links between 126 crops and forest tree species and 89 black-listed insect pest species, we show that the metawebs shift toward increased numbers of links and overlap of suitable area under climate change. Decomposing the metaweb across regions shows large saturation in southern Europe, while many novel interactions are expected for northern Europe. In light of the rising consumer awareness about human health and environmental impacts of food and wood production, the challenge will be to effectively exploit new opportunities to create diverse local agriculture and forestry while controlling pest species and reducing risks from pesticide use.

## Abstract (web summary)

Marc Grünig et al. report a study of land-use opportunities and risks of introducing novel insect pests in Europe that may arise from global climate change. Using a metaweb approach, they find that there is a predicted general increase in risk of pests to managed plant species under climate change due to an increase in land with suitable climate for both pests and plants.

## Introduction

Global changes, including biological invasions and climate change, have already affected human-managed ecosystems^1^ and are expected to continue to shape the productivity and diversity of agricultural and forest landscapes^2–5^. Agricultural and forest systems provide a variety of food and manufacturing resources, which are central to the functioning of societies^6–8^. European agriculture currently strives towards more sustainable management practices, including enhanced local food production and reduced use of pesticides^9,10^. Climate change might oppose these trends, and the design of innovative management practices will require adaptations to new environmental conditions^11^. Agriculture and forestry are particularly sensitive to abiotic changes^12^. Climate change may increase the productivity of crops and forest trees, e.g., via positive responses to higher CO_2_ concentrations^13^, but also increase yield losses from pests and pathogens^14–16^. Investigating future opportunities for crop cultivation and forest management under impending new threats from pest species is therefore crucial for addressing risks and opportunities in the agricultural and forestry sectors associated with future climate change.

At the global scale, climate change is expected to decrease crop production and hamper food security^17,18^. However, in some areas of Europe climate change may enhance productivity and provide opportunities for diversifying agriculture and forestry^19,20^. Like their natural counterparts, agricultural crop species and managed forest trees display an ecological niche of climatic preference^21^, and the suitable area for growth is expected to shift with increasing temperatures^22^. Particularly in northern regions, new opportunities for intensifying agricultural and forest resource utilization are predicted for the future^12,23^. Cropping area is expected to expand towards higher latitudes, raising productivity in Northern Europe^24^. Moreover, positive in situ effects, such as a prolonged growing season and increased CO_2_ fertilization, could boost the productivity of agricultural systems^24^ and forests alike^25,26^. For instance, the distribution range, production, quantity and quality of grapevines have been projected to benefit from climate change, thanks to higher CO_2_ concentrations^27^. Overall, the beneficial effects related to climate change are expected to provide new opportunities for crop and forest tree species in some European regions, but these gains might be counteracted by greater risks from climate extreme events^26^ and pest pressure^28^.

Insect pests already inflict major costs to the agricultural and forestry sectors, and their impact is predicted to increase under climate change^14,15,29^. Native and recently introduced alien insect pest species cause major costs to agricultural and forest production annually^30,31^. Pre- and post-harvest yield losses can each sum up to 10–16% of total annual crop production^30^. The extra pressure from invasive pests associated with the globalization of trades is expected to increase these costs further^32^. Whereas in the past the movement of species through commercial networks and their establishment in new regions was hampered by climatic barriers^33^, future climate change might lift abiotic barriers and enable the proliferation and spread of species^22^. In addition, milder winters will enable increased survival of more insect species at higher latitudes^29,34^. Following recent warming and globalization, the number of newly established alien species, including insect pests, has been rising in Europe^35–37^. For instance, the polyphagous fruit pest *Drosophila suzukii* has successfully colonized Europe, and is already causing large financial losses to growers^38^. In contrast to many native pests, for which effective management practices are in place, invasive pests require the deployment of new, still largely underdeveloped control measures. Anticipating the arrival of new pest species and understanding their interactions with crops and managed forests is crucial for designing management strategies for different invasion scenarios.

Here, we adopt a metaweb approach^39,40^ to study the present and future links and exposure of managed plants with their novel pests under climate change. We expect that climate change will promote: (1) new opportunities for cropping and forest systems owing to an increase of areas with suitable climate for growing more diverse crops; (2) higher pest pressure caused by increasing feeding interactions from novel invasive pests on managed plants (increasing number of links), (3) greater risks caused by larger overlaps of climatically suitable areas for host plants and their pests (increasing exposure). We forecast future climatic suitability for 96 economically relevant crops and 30 forest tree species from Europe and 89 insect pest species included in lists of the European Plant Protection Organization (EPPO). The considered pests are either recommended by experts to be regulated as quarantine species or have been recently identified as posing a risk to the EPPO region (www.eppo.int). We use species distribution modelling (SDM) and future climate scenarios in high spatial and temporal resolution to forecast climatically suitable areas for all species. We investigate the potential for plant growing under climate change within five categories (“fruit crops”, “vegetable crops”, “arable crops”, “other crops” and “forest trees”). Coupling the metaweb with forecasted climatically suitable areas, we predict how the linkage properties between host plants and pests, and the plant species exposure are affected by climate change. We further quantify pest pressure, as the number of pests with suitable climatic habitat, for five categories of pests (“fruit pests”, “vegetable pests”, “arable crop pests”, “polyphagous pests” and “forest pests”).

## Results

### Changing area of suitable climate for crops and forest trees

We predict that the area of suitable climate will increase for most crops and forest tree species within Europe between 2020 and 2100. We estimate a median increase in the area with suitable climate for crops from 1,925,265 km^2^ in 2020 to 2,790,484 km^2^ (+47%) under the representative concentration pathway (RCP) 8.5 and 2,487,919 km^2^ (+27%) under the RCP4.5 scenario in 2100. For forest tree species, the median area of suitable climate increases from 4,225,050 km^2^ in 2020 to 4,366,851 km^2^ (+3%) under the RCP8.5 scenario, less than forecasted under the RCP4.5 scenario (4,561,816 km^2^) (+8%) until the end of the century, because of the smaller loss in southern Europe.

**Fig. 1:**
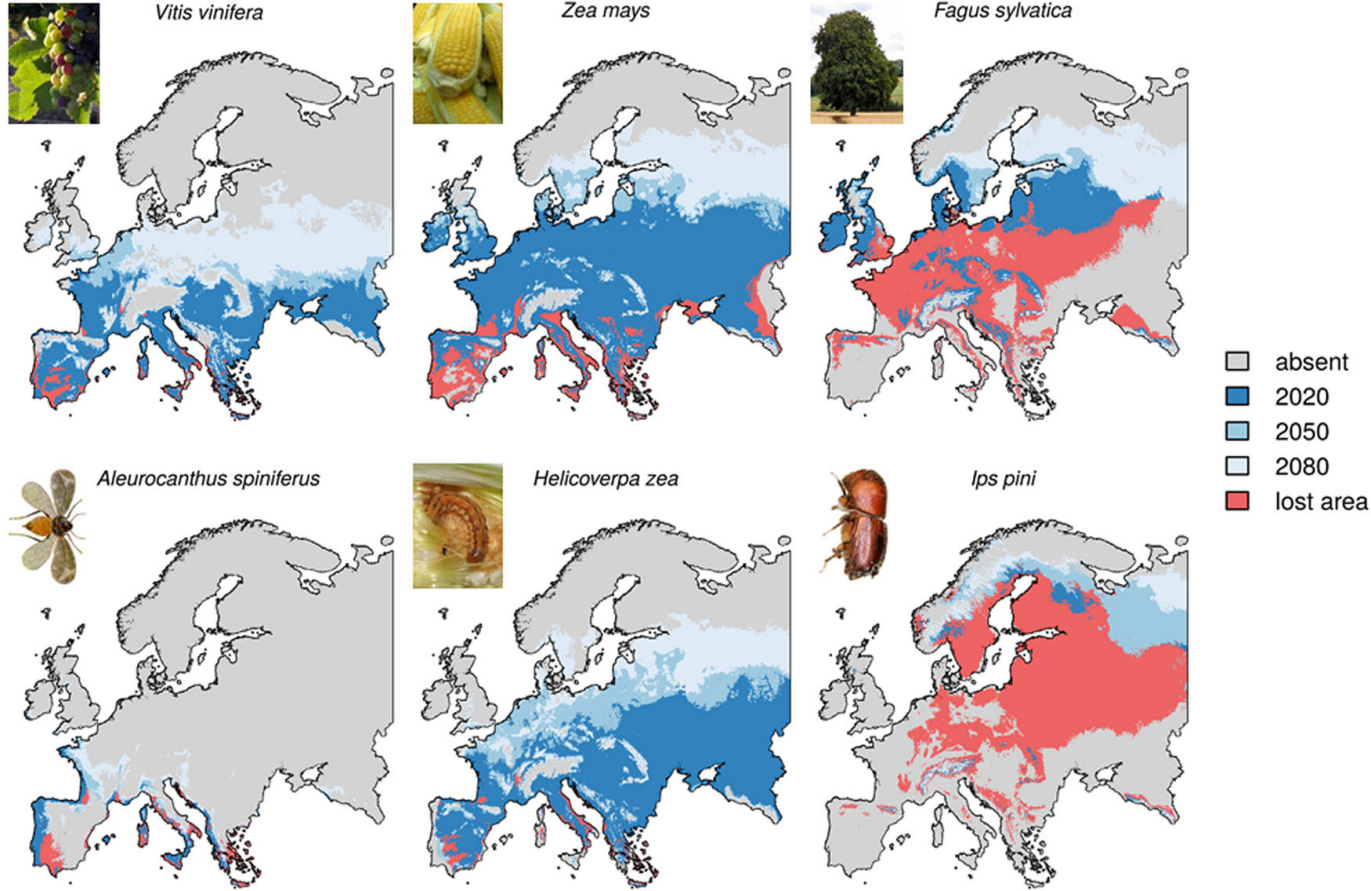
Predicted shifts of climatic suitability for exemplary host plant and insect pest species. Grapes (*Vitis vinifera*), maize (*Zea may*s) and European beech (*Fagus sylvatica*) show shifting climatic suitability towards higher latitudes. *Aleurocanthus spiniferus*, a fruit pest, *Helicoverpa zea*, an arable crop pest and Ips pini, a forest pest, also show northwards shifting climatic suitability under future climate conditions. Europe’s current climate already provides suitable conditions for these pests. Red areas show climatic suitability loss from 2020 to 2100. Together the red and the dark blue area show the modelled distribution in 2020. Projections under the RCP8.5 scenario are shown here. Images are licensed under the Creative Commons Attribution 3.0 Unported (https://creativecommons.org/licenses/by/3.0/deed.en/: Vitis vinifera: https://commons.wikimedia.org/wiki/File:Grapes_during_pigmentation.jpg; Ips pini: https://commons.wikimedia.org/wiki/File:Ips_pini.jpg), or the Creative Commons Attribution-Share Alike 2.0 Generic (https://creativecommons.org/licenses/by-sa/2.0/deed.en; *Zea mays*: https://commons.wikimedia.org/wiki/File:Mahane_Yehuda_Market_(9629714152).jpg; *Fagus sylvatica*: https://commons.wikimedia.org/wiki/File:Beech_(Fagus_sylvatica)_(19185865168).jpg; *Helicoverpa zea*: https://commons.wikimedia.org/wiki/File:Helicoverpa_zea_larva.jpg.) The image of *Aleurocanthus spiniferus* was offered as copyright free on http://www.ces.csiro.au/aicn/name_s/b_164.htm. All images were cropped to the fitting extent but remained otherwise unchanged.

Assuming a relationship between economic profit and climatically suitable areas, we predict increased cultivation opportunities for 82 (RCP8.5), respectively 91 (RCP4.5) out of 126 crop and forest tree species. Some of these species will have largely increased suitable climatic area and thus scope for growing economic significance. For example, in Europe soybeans currently have a gross production value of roughly 3.5 billion dollars (FAOSTAT^41^) and their suitable climate area will increase by 190% under the RCP8.5 scenario (95% RCP4.5) by the end of the century. We also predict an increase in the suitable area for many specialty crops with high market values (e.g., RCP8.5: apple +29%, grapefruit +756%, lemon lime +105%, melon +87%, tomato +42%; RCP4.5: apple +47%, grapefruit +225%, lemon lime 70%, melon +50%, tomato +23%). Meanwhile, for other economically relevant crops, the suitable climatic area within Europe is forecasted to decline substantially by 2100 under the RCP8.5 scenario (wheat −9%, maize −14% (Fig. 1), oats −44%, rye −76%, potatoes −20%), while more limited under RCP4.5 (wheat +4%, maize +7%, oats −9%, rye −28%, potatoes +1%). Forest tree species are predicted to lose suitable climatic area under steeper temperature increase (RCP8.5 *Abies alba* −73%, *Fagus sylvatica* −12% (Fig. 1), *Picea abies* −77%), but slower decline under the RCP4.5 (*A. alba* −36%, *F. sylvatica* +8%, *P. abies* −39%). We investigated the geographic differences in the change of climatically suitable areas. Our results for the five European regions highlight that new opportunities for the exploitation of crops and forest trees will open up, particularly in Northern Europe (RCP8.5: 48 species in 2020, +33 in 2100; RCP4.5: 48 +16) and the British Isles (RCP8.5: 53 +28; RCP4.5: 53 +10). In Western (RCP8.5: 85 +9; RCP4.5: 83 +9) and Eastern Europe (RCP8.5: 90 +6; RCP4.5: 90 +7), slightly more species are expected to encounter suitable climate in the future, while in Southern Europe (RCP8.5: 101 −7; RCP4.5: 100 +0) the number of species with suitable climate is predicted to decrease.

### Increasing linkage between plants and pests

**Fig. 2:**
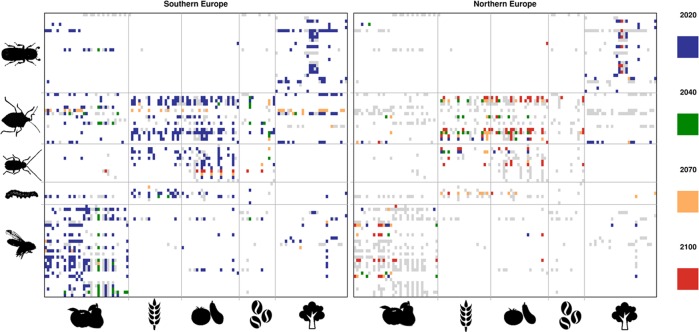
Predicted dynamics of realized interactions between insect pests and their host plants for Southern (left), and Northern (right) Europe under the RCP8.5 scenario. The interaction network for Southern Europe shows that most interactions are already possible under current climate conditions. Icons show different categories of pests (from bottom to top: “fruit pests”, “arable crop pests”, “vegetable pests”, “polyphagous pests” and “forest pests”) and host plants (from left to right: “fruit crops”, “arable crops”, “vegetable crops”, “other crops” and “forest trees”). “Fruit pests”, “polyphagous pests” and “forest pests” face the highest risk. Coloured points show the time step of first potential overlap between each pair of host plant and pest. In contrast, the interaction network for Northern Europe shows that many interactions become realizable only in the second half of the current century not until 2100 (grey links). Interaction networks for other regions and RCP4.5 scenarios are shown in Supplementary Figs. 1–5. Icons are pictures licensed under the Creative Commons CC0 1.0 Universal Public Domain Dedication (https://creativecommons.org/publicdomain/zero/1.0/deed.en), the Creative Commons Attribution-Share Alike 2.0 Generic licence (https://creativecommons.org/licenses/by-sa/2.0/deed.en), Creative Commons Attribution 3.0 Unported (https://creativecommons.org/licenses/by/3.0/deed.en) or Creative Commons Attribution-Share Alike 4.0 International (https://creativecommons.org/licenses/by-sa/4.0/deed.en). Images are available at the following URLs: Forest pests: https://commons.wikimedia.org/wiki/File:Lymantor_coryli_(Perris,_1855)_Syn.- Triotemnus_coryli_(Perris,_1855)_(15286593562).png; Polyphagus pests: https://commons.wikimedia.org/wiki/File:Halyomorpha_halys_s2a.jpg; Vegetable pests: https://commons.wikimedia.org/wiki/File:CSIRO_ScienceImage_7410_A_larva_of_Helicoverpa_armigera_the_worlds_worst_insect_pest.jpg Arable crop pests: https://commons.wikimedia.org/wiki/File:Diabrotica_virgifera_LeConte,_1868.jpg; Apple: https://commons.wikimedia.org/wiki/File:Manzana.svg; Pear: https://commons.wikimedia.org/wiki/File:Pear_icon.png; Arable crop: https://commons.wikimedia.org/wiki/File:Agriculture_-The_Noun_Project.svg; Tomato: https://commons.wikimedia.org/wiki/File:Twemoji_1f345.svg; Eggplant: https://commons.wikimedia.org/wiki/File:Twemoji_1f346.svg; Coffee: https://commons.wikimedia.org/wiki/File:Coffee_beans_by_gnokii.svg; Forest trees: https://commons.wikimedia.org/wiki/File:Noun_883674_cc_Symbolon_tree_icon.svg.

**Fig. 3:**
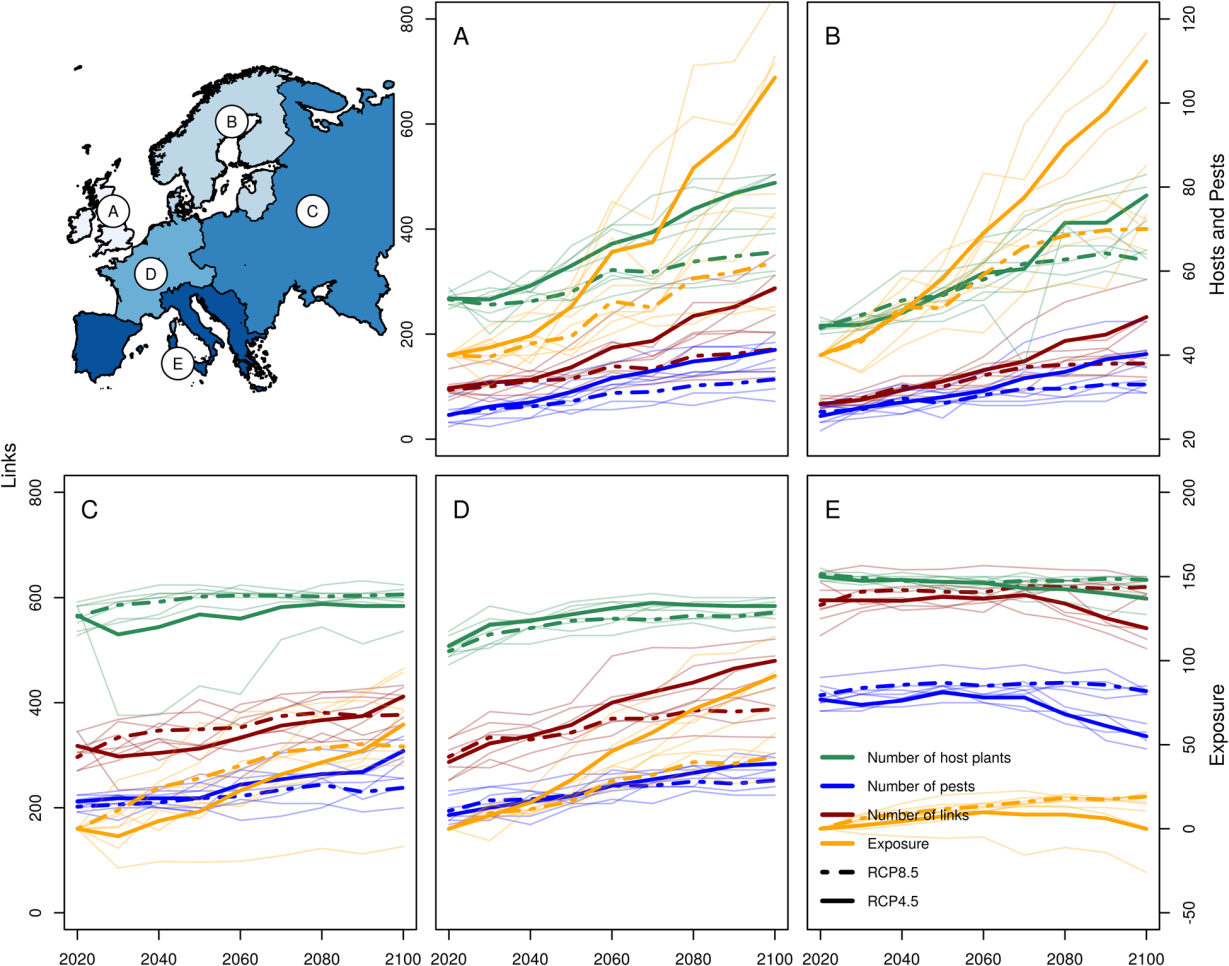
Network properties for European regions. European regions (**a** British Isles; **b** Northern Europe; **c** Eastern Europe; **d** Western Europe; **e** Southern Europe; upper left). The graphs show properties derived from metawebs of different regions. The number of links (red), exposure, as the mean overlap area per link in percent increase (orange), host plants (green) and pests (blue) with suitable climate conditions in 5000 grid cells or more are shown per time step (2020–2100) for all regions (**a**–**e**). Scales for the links are shown on the left side, for host plants and pests on the right side on the upper panels, for exposure on the right side on the lower panels of the figure. Thin lines show the results of the single GCM predictions and the thick lines the medians of the four GCMs per scenario. Additional metrics (specialization, modularity, increasing partner diversity and increasing number of shared partners) for host plants and pests are shown in the supplementary material (Supplementary Fig. 6).

We built a metaweb recording all known interactions between host plants and insect pests for Europe. We constrained the metaweb with potential range suitability overlap in order to quantify general changes in the incidence of pests on crops, under current and future climate (Fig. 2). The measured overlap of modelled climatic suitability of host plants and pests indicates increasing number of links and exposure (as mean overlap area per link) for Europe. The metaweb filtered by suitability overlap under climate change indicates that by 2100, up to 80% (RCP4.5: 79%) of links are predicted to be possible, notwithstanding large variation among regions (i.e. Southern Europe, Western Europe, Northern Europe, Eastern Europe, British Isles; Fig. 3) and time periods (2020–2100). In Southern Europe, 64% (RCP4.5: 63%) of the links can already be realized under the current climate. In contrast, in Northern Europe currently only 7% (RCP4.5: 7%) of the links can currently be realized. This leaves a large potential for increase by 2100, when up to 25% (RCP4.5: 15%) of all links become possible. Most interactions in Europe affect “fruit crops” (RCP8.5: 251 +11; RCP4.5: 244 +31) and “forest trees” (RCP8.5: 176 -4; RCP4.5: 171 +8). Economically relevant crops will be affected by many more potential pest species in Northern Europe towards the end of the century (maize: +7 links under RCP8.5/ +3 links under RCP4.5; wheat +4/+3, potato +6/+3, grapevine +5/+4). We find a stronger increase in links per pest species in Northern regions. The numerous links illustrate that Southern (RCP8.5: 595; RCP4.5: 586), Western (RCP8.5: 287; RCP4.5: 297) and Eastern Europe (RCP8.5: 318; RCP4.5 297) are already potentially threatened by pest invasions under the current climate. In contrast, Northern Europe (RCP8.5: +166 links; RCP4.5: +77) and the British Isles (RCP8.5: +190; RCP4.5: +78) show a strong increase in network links under climate change, indicating that crop diversification will come at the cost of higher pest pressure. While in Southern Europe, the number of links per species decreases on average from 6.7 to 6.1, it strongly increases in Northern Europe (0.8 to 2.6) and the British Isles (1.1 to 3.2). The predicted greater occurrence of generalist pests, such as the polyphagous *Spodoptera frugiperda* and *Helicoverpa zea* contributes to the rising number of links and links per species under climate change. While in Southern Europe the links with most of their host plants are already possible under current climate, in Northern Europe, the number of links will drastically increase for both of them (*S. frugiperda*: +19 links from 2020 to 2100 under RCP8.5, +10 RCP4.5; *H. zea*: +21 RCP8.5, +14 RCP4.5). The increase in the incidence of generalist species further causes a decrease in network specialization under climate change. In addition, the observed modular structure is predicted to be disrupted over time, where more generalist pests will attack different categories of host plants. These trends are supported by other network metrics such as increasing partner diversity and increasing number of shared partners for host plants and pests (Supplementary Fig. 6).

### Area of suitability overlap increases with climate change

Coupling the metaweb with projected climatic suitability indicates shifts in the exposure of managed plants to pests over time (Fig. 3). The mean area of overlap per link of the modelled climatic suitability of host plants and pests is predicted to increase by 51% (RCP4.5: 38%) between 2020 and 2100 in Europe. The exposure increases most in Northern Europe (RCP8.5: 173%; RCP4.5: 75%) and the British Isles (RCP8.5: 165%; RCP4.5: 57%). Also for Western (RCP8.5: 90%; RCP4.5: 43%) and Eastern Europe (RCP8.5: 60%; RCP4.5: 49%), we observe a marked increase in contrast to Southern Europe, where we predict little changes (RCP8.5: 0%; RCP4.5: is 19%). For instance, the increasing climatic suitability for some pest species promotes a marked increase of potential overlapping area with their host plants (*S. frugiperda*: +176% RCP8.5; +70% RCP4.5; *H. zea* +88% RCP8.5; +42% RCP4.5). For different categories of host plants, we find similar patterns of slightly increasing area of overlap in all regions but Southern Europe, where we predict decreasing area of overlap for forest trees and arable crops (Supplementary Figs. 7 and 8). For pest categories, we predict that forest pests will overlap less with their host plants in the future, while all other categories will have larger overlap of suitable areas towards the end of the century (Supplementary Figs. 9 and 10). Although the climatic suitability drops for some crops, the area of overlap of these crops with their pests is nevertheless predicted to increase, as found for maize (RCP8.5: +110%; RCP4.5: +39%), wheat (RCP8.5: +135%; RCP4.5: +40%) and potatoes (RCP8.5: +80%; RCP4.5: +44%).

### Changing area of climate suitability for pests

**Fig. 4:**
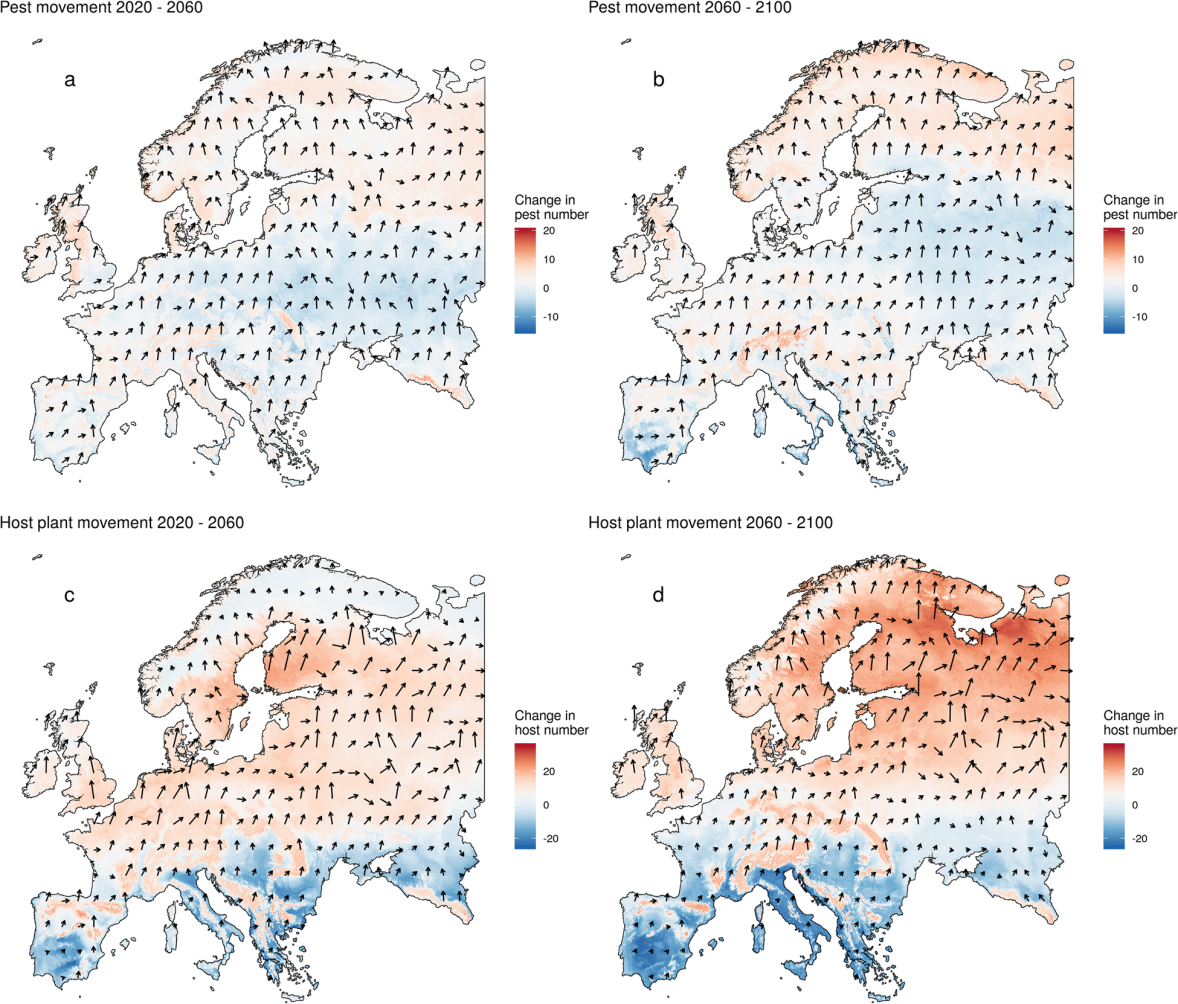
Climatic suitability shift for pests and host plants. Arrows show for each grid cell the average direction of climatic suitability shift over all species. For each species, we calculated the direction from where each newly suitable grid cell can be reached from its closest suitable grid cell in the previous time step. The length of the arrows is proportional to the number of new colonisations of each grid cell. The coloured maps show the change in total number of pests (top) and host plants (bottom) with suitable conditions during the time steps of 2020–2060 (left) and 2060–2100 (right). Red shadings indicate an increase of the number of species with suitable climate; blue shadings indicate decreasing numbers. Climatic suitability shift and change in number of species are shown for the RCP8.5 scenario (see Supplementary Fig. 11 for RCP4.5).

Overall, the median area with suitable climate for insect pests (2,491,321 km^2^ in 2020) will increase under climate change associated with a northward expansion of pest species. We forecast an average increase in the suitable area for pest species of 294,176 km^2^ (+12%) under the RCP8.5 scenario and 229,981 km^2^ (+9%) under the RCP4.5 scenario. Most of the considered pests already have suitable climatic conditions in Europe. In particular, Southern Europe (RCP8.5: 71; RCP4.5: 71) is already threatened by many of the pests on the EPPO lists. In Northern Europe (RCP8.5: 26 +14; RCP4.5: 27 +5), Western Europe (RCP8.5: 43 +13; RCP4.5: 44 +8), Eastern Europe (RCP8.5: 47 +11; RCP4.5: 45 +6) and the British Isles (RCP8.5: 25 +17; RCP4.5: 26 +9), pest pressure will increase until 2100 (Fig. 3). Under the RCP8.5 we predict increasing suitable climate area for 60 of 89 pest species (71 under RCP4.5), and hence important potential expansions such as for the fall armyworm (*S. frugiperda)*, which will increase by 81% (3,341,038 km^2^ under current conditions; 51% under RCP4.5), corresponding to additional 2,758,535 km^2^ (1,676,514 km^2^ under RCP4.5). We quantified the dynamic shifts of climatic suitability for host plant and pest species from and to each colonized grid cell in Europe between 2020–2060 and 2060–2100 (Fig. 4). The shift of climatic suitability for host plants shows a gradient towards higher latitudes, underlining the opportunities arising in northern regions in the second half of the century. Meanwhile, the shift of climatic suitability for insect pest shows no clear south-north gradient, possibly because of more complex and diverse climatic niche shapes of pest species. Consequently, the dynamic of the shift of insect pest species is expected to be more idiosyncratic than that of their host plants under climate change. We observe a slight decrease of the number of pest species in central and northeastern regions, caused by the gap in climatic niches between cold-adapted pests and more warm-adapted pests (Supplementary Fig. 12). While cold-adapted species will move further north with increasing temperatures, warm-adapted species are lacking behind. Finally, we analyzed the shift in centroid position of all modelled ranges of host plants and pest species by measuring the direction and distance of the movement between 2020 and 2100 (Supplementary Figs. 13 and 14). The centroid analysis shows a median distance of 519 km, and speed of 6.5 km/year for pests under RCP8.5 (240 km; 3.0 km/year for RCP4.5), values that are consistent with published estimates of dispersal capacity^42^. The analysis indicated a median distance of 588 km for host plants, resulting in a speed of 7.3 km/year (269 km; 3.4 km/year for RCP4.5).

## Discussion

The metaweb approach adopted in our study indicates a general increase in susceptibility of managed plants to pests under climate change owing to (1) an increase in the number of links between crops, forest trees and their pests, and (2) an increase in the area of climatic suitability overlap between pests and plants, which will challenge the benefit of climate change on agricultural diversification. In contrast to the increase in potential distribution for single crops (e.g., for maize^43^), here we show a general pattern of increasing climatic suitability for a wide variety of crops and forest tree species, indicating that climate change will favour diversification of European plant production across different subsectors. While in Southern Europe future climate will become increasingly unsuitable for staple crops like wheat, maize and potatoes, future suitable areas are identified in northern countries, partly offsetting the loss in the South. In northern European regions, the potential for growing more valuable crops and trees provides scope for enhanced economic profit. For example, grapes are currently harvested on 3,429,137 ha in Europe, resulting in a gross production value of roughly 30 billion dollars (FAOSTAT^41^). We forecast that the suitable climate for growing grapes will increase by 136% under the RCP8.5 (71% under RCP4.5) by 2100. If the production area increases in proportion, gross production values could increase by roughly 22–40 billion Euro. While the agronomic and economic implications are far more complex, we highlight a wider range of opportunities for growing crops across Europe under climate change. Since multiple crops cannot spread simultaneously into new areas, the decision of realizing new crop potential will depend on the market prices, consumer demands, regulatory frameworks and cultivation decisions at the farm level.

Increasing climatic suitability and associated positive effects for host plants might be offset by simultaneously increasing number of links between managed plants and pests at their degree of exposure. By quantifying changes in interactions between pests and their host plants under climate change within a metaweb, we demonstrated that increasing plant climatic suitability is accompanied by increasing pest pressure across Europe. Using a metaweb including 89 pests and 126 host plants allows us to investigate the change in the system as a whole, including a variety of climatic niches (Supplementary Fig. 12) and all European regions. We show how the web of agricultural pests and cultivated plants is forecasted to change, rather than predicting the suitable climate for single pests and crops e.g.^43,44^. We found that polyphagous pests will most expand the interaction area with their host plants, while for forest trees the exposure to pests decreases on average (Supplementary Fig. 9). Generalist species affecting crops will benefit most from warmer temperatures with a larger potential distribution and increasing number of possible links. This is underlined by several metrics of the interaction network (decreasing modularity and specialization, and increasing partner diversity and number of shared partners), showing that the average number of interactions per pest and the links of pests with other categories of hosts will increase in most regions, while specialization will decrease. The invasion success, and thus the nature and extent of pest threats, and the damage caused depend on host availability^45,46^, while larger areas of overlap between plants and pests have a larger potential for interactions and therefore larger population sizes and higher invasion risk^47^. We observe a sharp rise of exposure to pests, especially in Northern Europe and the British Isles. Although northern regions are predicted to benefit most from increasing climatic suitability for crops and forest tree species under climate change^19^, they will also become more targeted by pest invasions. Global changes in climatic suitability of pest species has been shown with correlative models, indicating higher pest pressure in high latitude regions^48^, and rising pest pressure has been associated with increasing metabolic rates of pests and therefore increasing crop losses in warmer climates^14^. Our metaweb approach corroborates this trend, and adds a thorough quantification of the nature of the risks in terms of link distribution and their strength. Seizing new opportunities will require weighting the benefits of new exploitation opportunities against the costs of co-occurrence of the novel crop or tree species and their associated novel pest species, whereby the latter may also collaterally affect other host plants.

The general pattern of pest range shift to higher latitudes will likely be associated with increasing yield losses and pest management costs^48^. Our approach to model the change in climatic suitability with a high temporal resolution of climate change illustrates the potential direction and speed at which species can be expected to spread. We show that under current climate conditions, most species could invade parts of Southern and Western Europe, and from there spread north-east with the changing climate. The predicted median speed of 6.5 km/year for the RCP8.5 emission scenario (RCP4.5: 3.0 km/year) is well in line with previous estimates for invasive insect species. Roques et al.^42^ estimated spreading rates of accidentally introduced species of 3.5 km/year, but results varied widely among insect orders (e.g. 7 km/year for Coleoptera). Assuming that the EPPO lists are proportionately representative for the categories of pests in the pool of pest species, we predict that most interactions will occur for pests of “fruit crops”, “vegetable crops” and “forest trees”, indicating that these are the crop, respectively tree categories most jeopardized by pest invasions. The number of interceptions at European borders between 1995 and 2004 show that Hemiptera (sub-order Sternorrhyncha), Diptera and Coleoptera are the orders intercepted most often of all insect pests^49^. In our dataset, 60 species belong to these orders and 47 of these species are either “fruit pests”, “vegetable pests” or “forest pests” (Supplementary Data 1). This indicates that our selection reliably reflects current propagule pressure and that the number of included species allows representing these different categories adequately. Further, we point out the difference in predicted pest pressure between the two RCPs. The median area of the modelled distribution of pests and the median overlapping area under the RCP4.5 scenario increase much less than under the RCP8.5 scenario (Supplementary Fig. 12). Northern regions might therefore suffer from fewer pest invasions under the RCP4.5 than under the RCP8.5 scenario (e.g., Northern Europe: +14 RCP8.5, +5 RCP4.5; British Isles: +17 RCP8.5, +9 RCP4.5), thus corroborating the urgency of policies aimed at restricting CO_2_ emissions in the near future.

An alarming implication of our results is that in large parts of Europe (i.e., mainly Southern and Western Europe) many of the invasive pests included in our analysis can survive under current climate conditions. In these regions, many host plants of these invasive pests can already be grown, and most network links are thus feasible, highlighting that invasion risks are an impending reality with the potential to severely disrupt the ecology and economics of managed ecosystems. This finding underscores the urgency of rapidly deploying support to phytosanitary services in Mediterranean countries. Pathways of insect pest invasions are often associated with accidental introductions by international trade, cargo movement and individual travel^35,50,51^. Once introduced and established in a new region, a pest might spread further to other regions with suitable conditions. Interception statistics from cargo control show that live plant imports bear an especially high risk of transporting insects^52^. Phytosanitary services have strict regulations for the inspection and control of live vegetal goods^45,53^, which will become even stricter in the European Union under the Regulation EU 2016/2031. However, international trade and travel have reached such a large volume, that screening and inspecting all potential routes of invasions is no longer feasible^51^. Finally, we emphasize the importance of preparing for scenarios where pests overcome natural barriers by human-aided transport. As noticed before, Southern Europe is already an entry gate for many subtropical pest species such as *Aleurocanthus spiniferus* (Fig. 1).

In conclusion, we showed that the structure of the plant-pest metaweb will be altered under climate change, favouring greater diversity of managed plants and incidence of pests, especially of generalist ones. In Europe, climate change could overall have beneficial effects on the diversity of crop production. However, to exploit this potential, it is crucial to monitor and prepare for potential collateral risks of pest pressure. Pest pressure presents a severe threat to European agriculture and forestry already under the current climate and will keep rising in the future. Reaping the benefits from the newly arising opportunities while minimizing the costs associated with the risks of climate change requires strong efforts and collaborations among all stakeholders in the food and wood production chains.

## Methods

### Data collection

We considered all crops for which distribution ranges are available from Earthstat^54,55^ (www.earthstat.org) and economically important forest tree species of Europe. We downloaded distribution ranges for crops as raster from Earthstat^54,55^ and for forest trees as shapefiles from EUFORGEN (www.euforgen.org) whenever available and presence records from GBIF (www.gbif.org) for all other species. We only included crops and forest trees listed as host plants for at least one of the pest species included in the EPPO plant quarantine lists (Alert, A1, A2; www.eppo.int). Vectors of plant pathogens were not considered. Occurrence records for pests were collected from various databases and from the published literature (see complete list of host plants and pests in Supplementary Data 1 and 2). We considered only species reported as present in fewer than five European countries in order to abstract from species already established on the continent, and strengthen the focus on pests to be expected in the future. We did not consider occurrences from stepping stones such as greenhouses and other structures that provide protection from unsuitable climate and thus promote the proliferation and spread of invasive species by enabling them to bridge unsuitable conditions and build up early generations in spring (e.g., *Tuta absoluta*^56^). Coordinates of occurrence records were mapped for each individual species and checked for unreasonable records by comparing with EPPO PQR database (https://gd.eppo.int) distribution maps, which show for each country if a species is present or absent. To prevent from multiple records per cell and reduce sampling bias, we filtered the data with a minimum distance between each pair of records. With the remaining occurrence records the geographic extent of the species range is represented as reliably as possible (i.e., records in native and invasive range). To secure adequate SDM performance, we excluded species with fewer than 24 occurrence records (8 records per explanatory variable). In total, 128 host plant species and 94 insect pest species met these criteria.

### Species distribution modelling

SDMs were calibrated using ensembles (unweighted average) of four widely used modelling techniques (generalized linear models, generalized additive models, gradient boosting machine, random forest) or a subset. We used a pseudo-absence approach, which is widely recognized as a solution for overcoming the lack of species absence data^57^. For each species, we randomly sampled 5000 pseudo-absences from biomes in which the species’ occurrence records lay. We down weighted the pseudo-absences to reach a prevalence of 0.5. In a first step, we projected the models globally to check the potential distribution under current climate visually. For future projections, we projected our models only to Europe. To evaluate model performance, we used the area under the ROC-plot curve (AUC)^58,59^ and true skill statistics (TSS)^60^. We used a split sample approach (70% calibration data and 30% evaluation data) with 20 repetitions. Models were considered to have a reliable performance with AUC scores > 0.7^61^ and TSS values > 0.4^62^. Models with AUC < 0.7 were not included in the ensemble. Five pest species and two crop species with unsatisfying evaluation metrics were excluded from the analysis (see Supplementary Data 3–6 for model performances).

Using SDMs to model the climatic suitability of pests and plants is a common and widely accepted approach. However, we are well aware of shortcomings when applying SDMs to invasive species. Invasive species tend to occur in a broader climatic niche in their invasive range than in their native range, for example because of the lack of natural enemies. This may lead to an underestimation of the region of climatic suitability for pests when only the native climatic niche is modelled. To overcome this caveat, we covered the native and invasive range of pests by including distribution records from their entire known range whenever possible^63^ (see Supplementary Data 7 for more detailed description). Further, we omitted biotic factors and dispersal limitations in our models. While we can assess the climatic niche of the species, in reality their distribution may be constrained by these factors. For host plants, the soil properties are also a major restricting factor. Therefore, we expect to overestimate potential distributions and the changes in the network. However, this limitation should mostly impact forest species because the movement of crops and pest species is affected by agronomic decisions, and thus only partly dependent on dispersal abilities. Finally, irrigation has a large impact on the distribution of many crop species. As precipitation patterns will differ from current conditions under climate change, water scarcity may limit crop irrigation in many parts of Europe during growth periods, restricting the distribution of crops. However, these changes are difficult to predict and beyond the scope of this study. Here, we addressed the issue by comparing models including both temperature and precipitation variables with models based on temperature alone. We found good agreement between the two approaches and hence applied SDMs based on only temperature variables to crops.

### Climate data and climate change scenarios

For historical climate data, we used the CHELSA V1.2 dataset^64^ (www.chelsa-climate.org) with a 2.5 arc min (~5 km) resolution. For future scenarios, we used model output statistics in combination with mechanistic downscaling (the CHELSA algorithm) to calculate mean monthly maximum and minimum temperatures, as well as monthly precipitation sums at a ~5 km spatial resolution globally for the years 2006–2100. Projected future climate variables were taken from four global circulation models (GCMs) driven by two scenarios of representative concentration pathways (RCP4.5 and RCP8.5) in a factorial manner. The four selected models originate from the CMIP5 collection of model runs used in IPCC’s 5th Assessment Report^65^. Different GCMs are, however, often based on similar code, and hence generate similar output^66,67^. We therefore chose models characterized by only a small amount of interdependence to allow for a good representation of uncertainty in climate projections. Model selection was based on model interdependence in ensembles^67^. Data were taken from the following four models: CESM1-BGC, run by the National Center for Atmospheric Research (NCAR); CMCC-CM, run by the Centro Euro-Mediterraneo per i Cambiamenti Climatici (CMCC); MIROC5, run by the University of Tokyo; and ACCESS1-3, run by the Commonwealth Scientific and Industrial Research Organization (CSIRO) and the Bureau of Meteorology (BOM), Australia.

We aggregated current climate data (1979–2020) in 5-year time intervals, from which we extracted climate data for all presence and absence records of pests, considering their sampling year if available. Records older than 1979 were coupled with the first time step. Records with no sampling year were coupled with an average of the historical data. We coupled host plant records with a baseline of future climate (2006–2020) for each GCM and RCP. In addition, we considered the resolution of the presence records. For low-resolution records (lower than 2.5 arc min), we extracted climate data from aggregated variable layers (5 arc min). Presence records with a precision of less than 5 arc min were excluded. For model projections, we aggregated time series of future climate (2011–2100) into 10-year time steps. For host plant SDMs, we used subsets of the following five explanatory variables: mean annual temperature, temperature seasonality, growing degree-days above 5 °C, annual precipitation and precipitation seasonality. In parallel, we ran models for all crop species without precipitation variables. Due to crop irrigation, precipitation might an unreliable predictor of the distribution of crop species. We tested both approaches and found very similar results. For the final analysis, we thus used models based on temperature alone. For pest SDMs, we chose the following variables: minimum temperature of the coldest month, growing degree-days above 5 °C, annual precipitation and precipitation seasonality. For 15 pest species, we added temperature seasonality to the explanatory variables and used a subset of the five variables to reach better model performance. All explanatory variables were chosen based on ecological significance^68,69^. We chose the variables based on ecological importance rather than statistical information criteria^70^ (see Supplementary Data 1 and 2 for the variables included for each species). Overall, we followed the recommendations to meet sufficient best practice standards of SDM^70^.

For further analyses, we applied a binary classification of the climatic suitability to each model output. We used the sensitivity-specificity sum maximization approach to define the threshold that separates suitable from unsuitable climate^71^ (R package *presenceAbsence* 1.1.9^72^). To apply binary classification to the ensembles, we used the average of the thresholds of all individual models included in the ensemble. Further, in all models we restricted the area of crop distribution with a cropland mask derived from Earthstat^54,55^. We analyzed the number of species per grid cell within Europe by overlaying binary model outputs of all species. We measured the area of suitable climate for each species as the sum of the specific area of all cells classified as suitable. For each European region (i.e., Southern, Western, Eastern and Northern Europe and the British Isles), we calculated the number of species with suitable climate conditions in at least 5000 grid cells (representing about 10% of the median agricultural surface of the different regions) per time step.

### Metawebs

We generated a metaweb^39,40^ recording all known interactions between pests and their host plants, which defines all possible network links. For each pest, we used a list of host plants given in the EPPO database on quarantine pests (http://www.eppo.int). We investigated all potential links for each time step by testing whether modelled distributions of host plants and pests overlap in at least 5000 grid cells. We did not consider host shifts that can occur in the invaded range, climate-driven evolutionary change, or phenotypic plasticity in either host plants or pests, their interactions and their interactions with other species^73^. We measured the area of overlap in the same way as the area of suitable climate (see above). For each European region, we counted the interactions that occur in at least one grid cell. To summarize the numbers from the different GCMs we calculated the median of each metric. We calculated exposure as the mean of the overlap area of all links for each time step. Modularity, specialization, partner diversity and number of shared partners were calculated with the R package *bipartit*e 2.13^74^.

### Climatic niches

Climatic niches of host plants and pests were computed based on the worldwide modelled distribution under current temperature. For each species, we isolated all grid cells predicted as suitable by our models and obtained the annual minimum temperature for these grid cells for current conditions. The range of occupied minimum temperatures was then used as the climatic niche over minimum temperature. For the borders of minimum temperature in Europe, we obtained the minimum and maximum value of the annual minimum temperature raster of Europe for 2020, 2060 and 2100 as a mean of the four GCMs.

### Spatial analysis of shift in climate suitability

To calculate the direction of shifts of climatic suitability, we extracted newly suitable grid cells for each species and all 10-year time steps. For each newly colonized grid cell, we identified the closest already occupied grid cell in the previous time step and measured the direction from there to the focal cell in a 25 km resolution. We averaged the direction for each grid cell over all species and counted the number of species colonizing each grid cell. We displayed the direction in a 250 km resolution to the highlight general regional patterns. Further, we averaged the direction and number of colonization of the single time steps for 2020–2060 and 2060–2100. We did not apply a crop mask to the modelled distribution of crops for this analysis because excluding grid cells led to unreliable averages during the interpolation of the direction. The analysis of the shift of climatic suitability is a qualitative representation to investigate general patterns and, therefore, small-scale inaccuracies might occur. Centroids were calculated as the latitudinal mean and the longitudinal mean (weighted with the cosine of latitude) of all occupied grid cells. All analyses were done in R version 3.5.1^75^.

### Statistics and reproducibility

For producing random numbers, we applied the *set.seed* function of the R package *base* (version 3.5.1)^75^ to enable reproducibility.

### Reporting summary

Further information on research design is available in the Nature Research Reporting Summary linked to this article.

## Supplementary information

Supplementary Information

Supplementary Data 1

Supplementary Data 2

Supplementary Data 3

Supplementary Data 4

Supplementary Data 5

Supplementary Data 6

Supplementary Data 7

Description of Additional Supplementary Files

Reporting Summary

## Data Availability

Climate data are available from www.chelsa-climate.org. Crops distribution maps available from www.earthstat.org, forest distribution maps from www.euforgen.org. Pest distribution records were gathered from published literature and databases are available from the corresponding author upon request. All source data underlying Figs. 1–4 are available in Zenodo with the identifier 10.5281/zenodo.3746103^76^.

## Code availability

The R codes for species distribution modelling, calculations of the results and analysis are available from 10.5281/zenodo.3746103^76^.
