# Supplementary material for: Crop and forest pest metawebs shift towards increased linkage and suitability overlap under climate change
Source: Commun Biol. 2020 May 11;3:233. doi: 10.1038/s42003-020-0962-9 (PMC7214431; doi:10.1038/s42003-020-0962-9)
Supplement: Supplementary file 1 — Supplementary Information [file 42003_2020_962_MOESM1_ESM.pdf]

Supplementary material

Supplementary figures

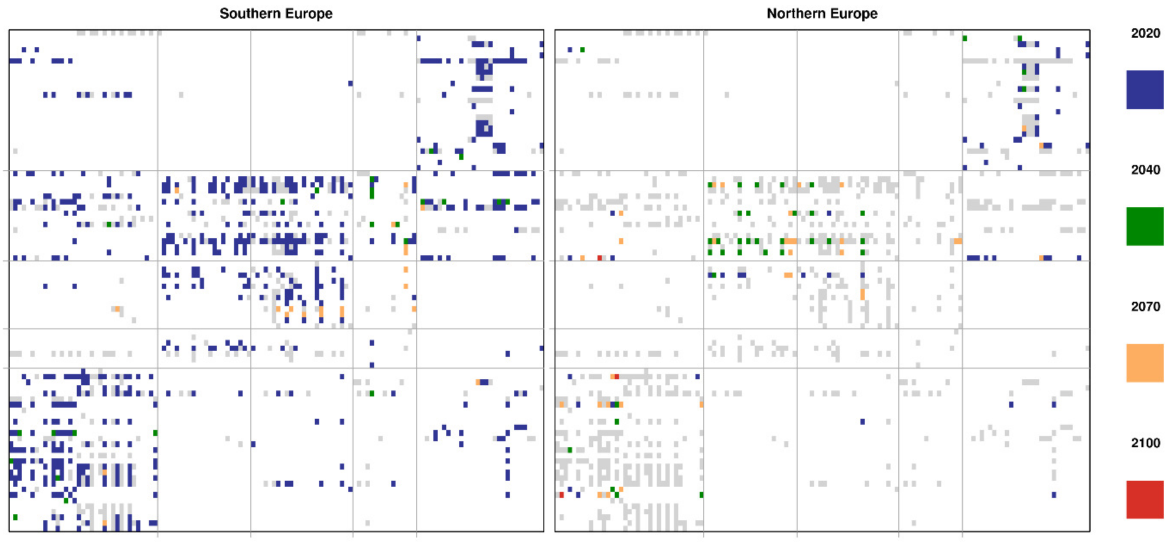

Supplementary Figure 1: Evolution 2020-2100 of the interaction network for Southern (left) and Northern Europe (right) under the RCP4.5 scenario. The geographic extent of the regions is defined in Fig. 3.

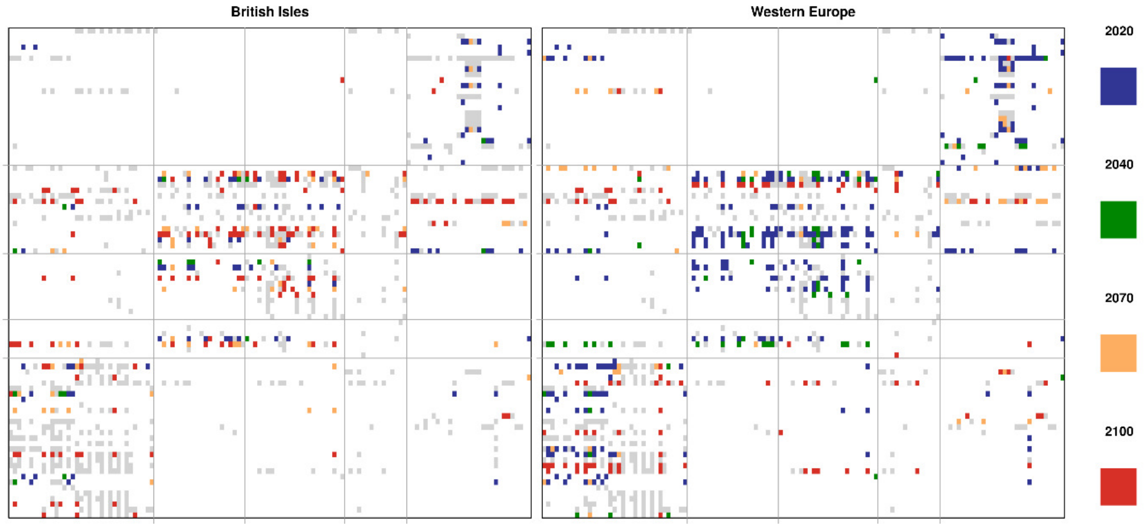

Supplementary Figure 2: Evolution 2020-2100 of the interaction network for the UK (left) and Western Europe (right) under the RCP8.5 scenario.

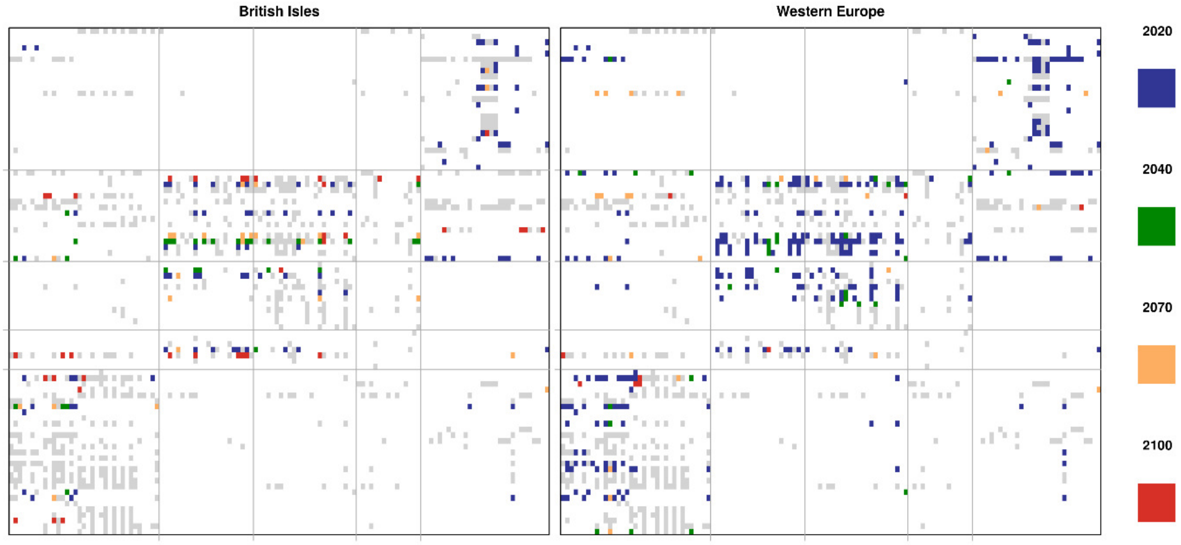

Supplementary Figure 3: Same as supplementary Fig. 2 but for the RCP4.5 scenario.

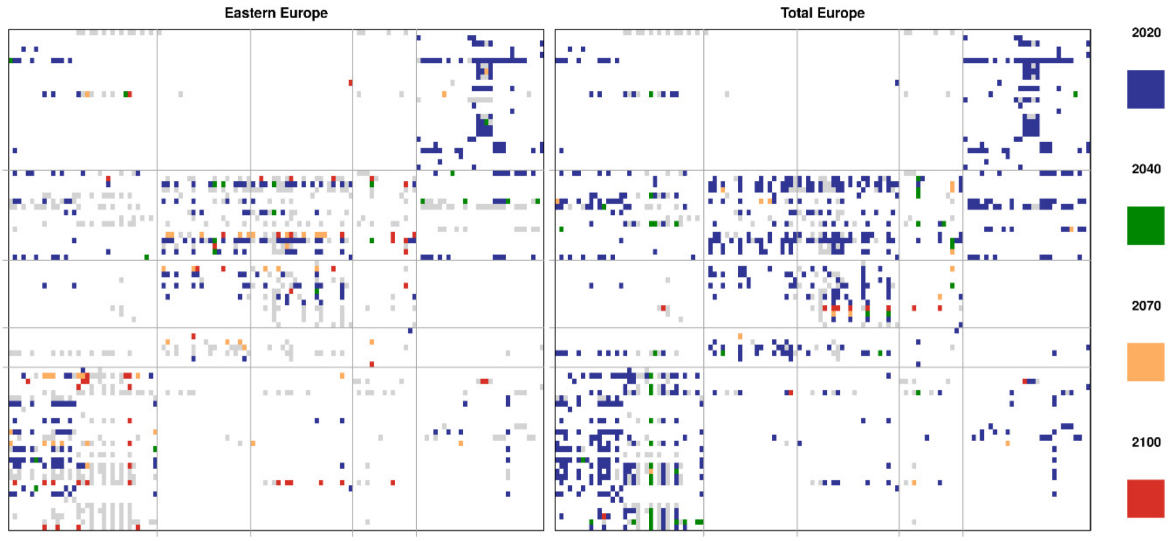

Supplementary Figure 4: Evolution 2020-2100 of the interaction network for Eastern Europe (left) and total Europe (right) under the RCP8.5 scenario.

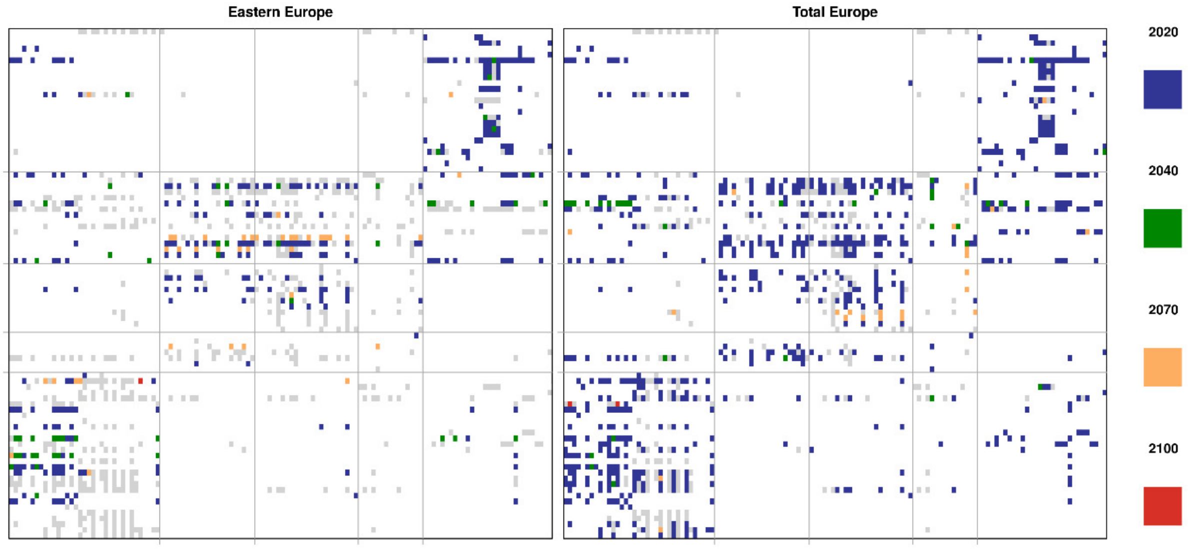

Supplementary Figure 5: Same as Fig. S4 but for the RCP4.5 scenario

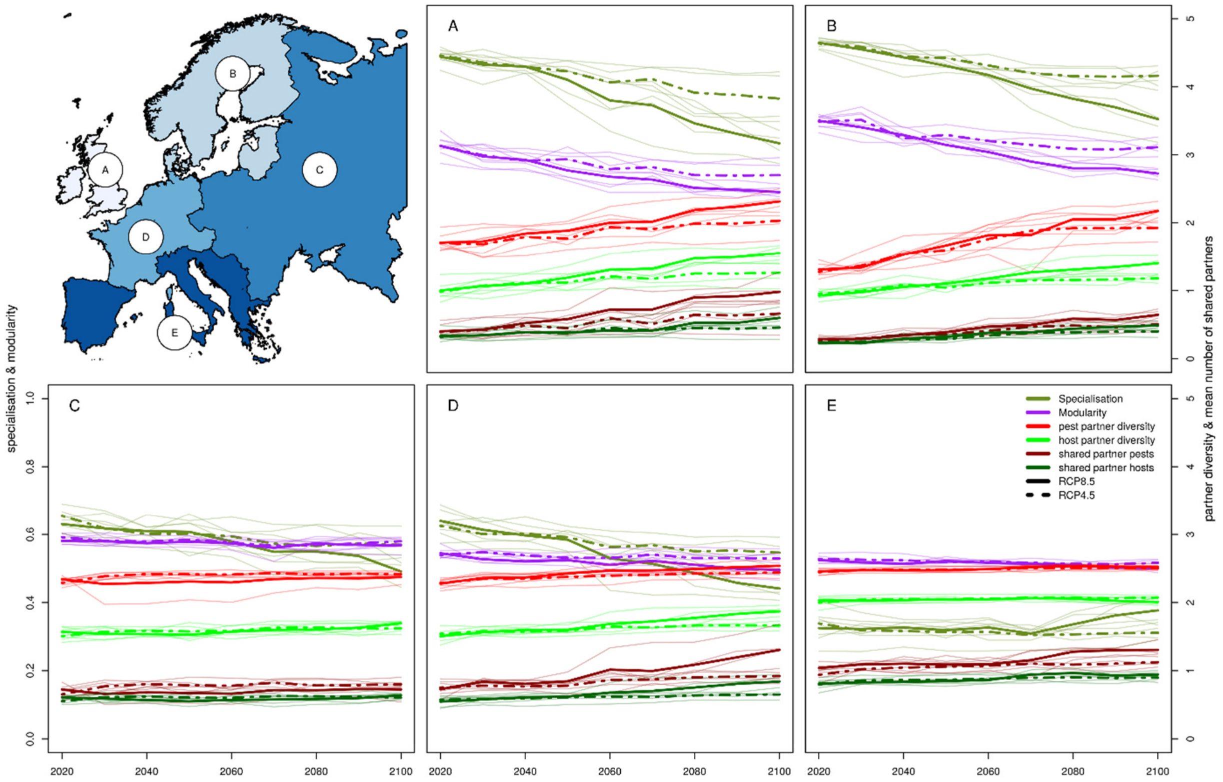

Supplementary Figure 6: Same as Fig. 3 but for modularity and specialisation. Modularity was calculated with the calculateModule function (R package bipartite 2.11). For specialization, we calculated the sum of all links for each pest species and used the mean of all sums as a metric.

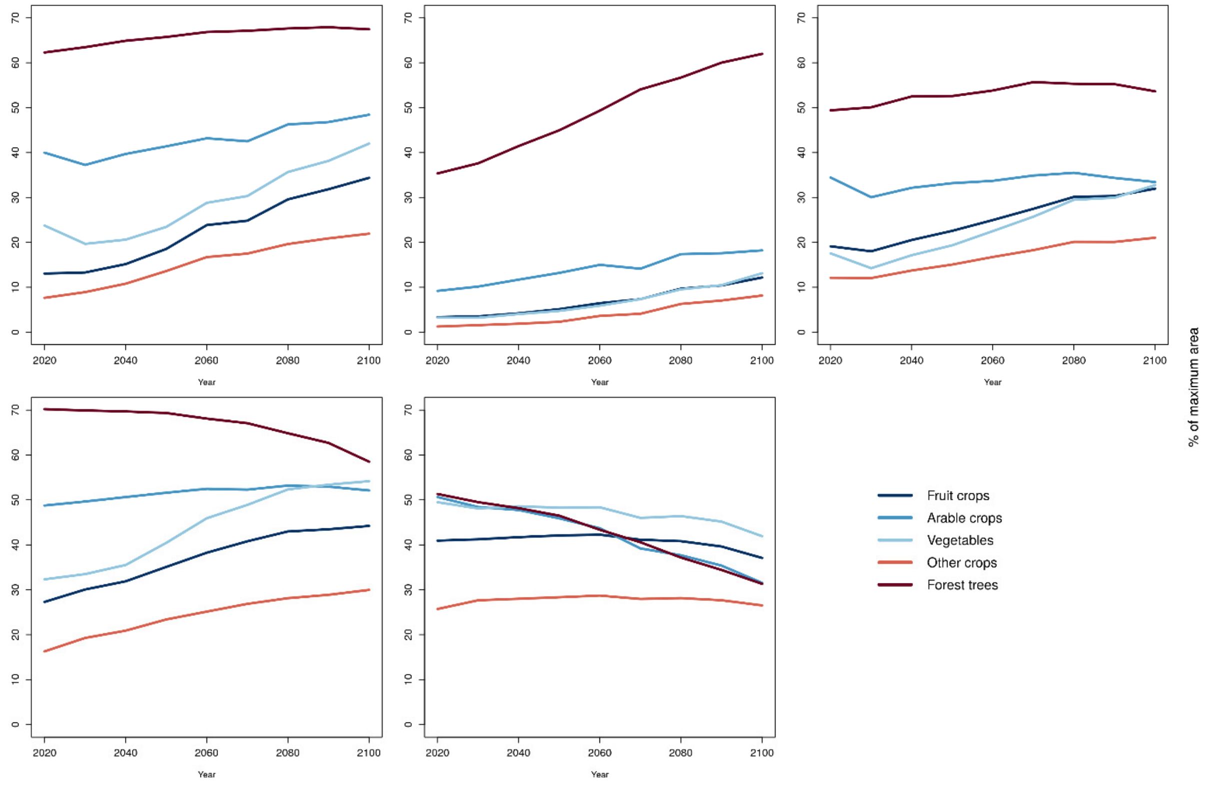

Supplementary Figure 7: Change in the area of overlap over time for the five different host categories under the RCP8.5 scenario. Panels from left top to right bottom: British Isles, Northern Europe, Eastern Europe, Western Europe, Southern Europe.

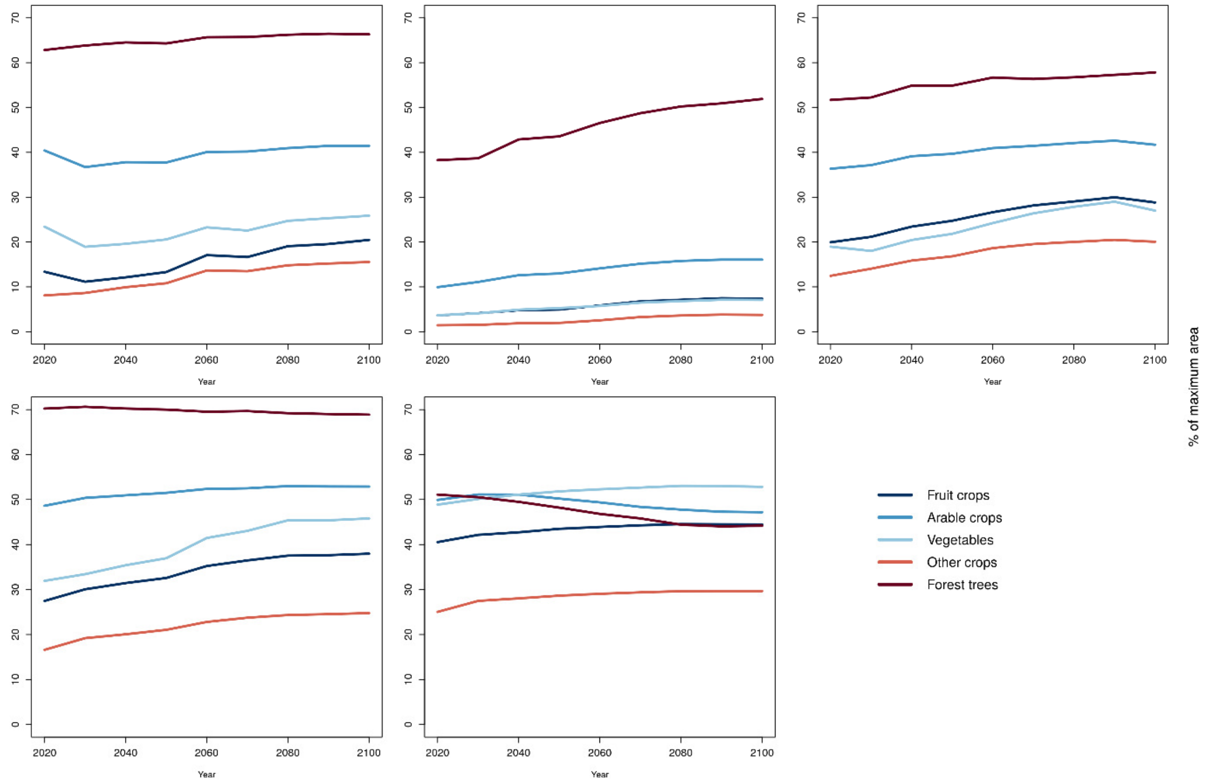

Supplementary Figure 8: Same as fig. S7 but under RCP4.5 scenario.

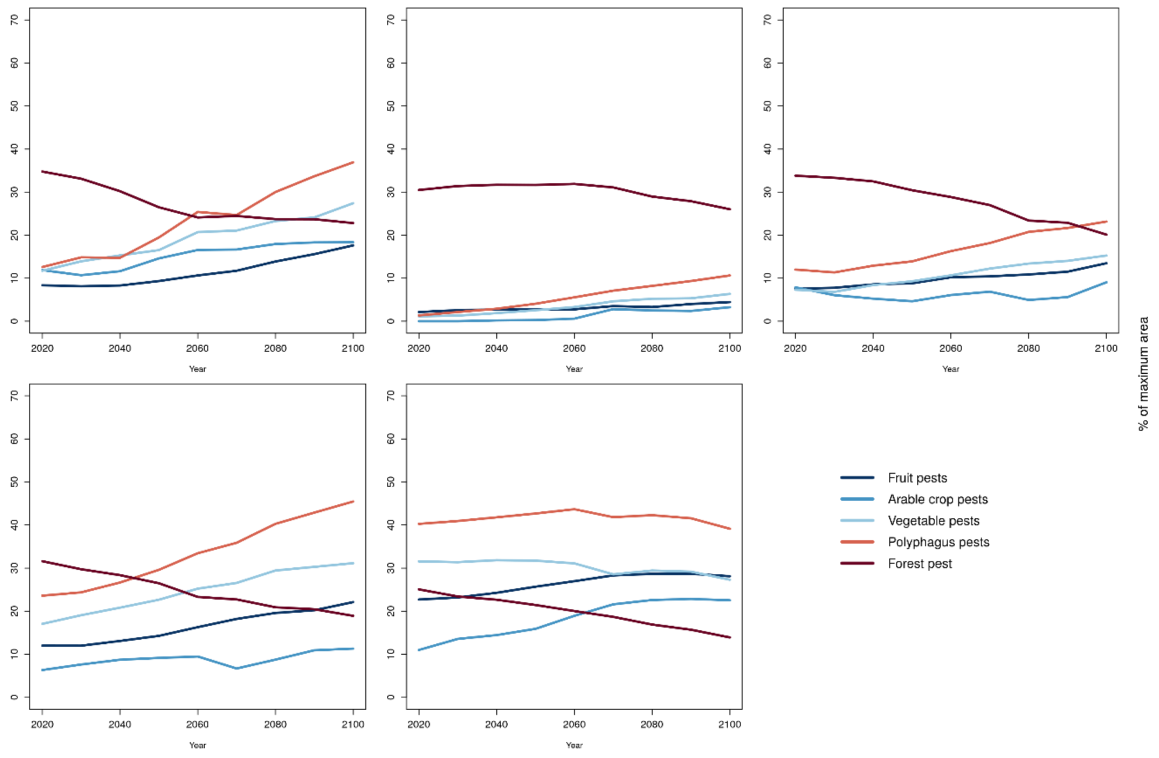

Supplementary Figure 9: Change in the area of overlap over time for the five different pest categories under the RCP8.5 scenario. Panels from left top to right bottom: British Isles, Northern Europe, Eastern Europe, Western Europe, Southern Europe.

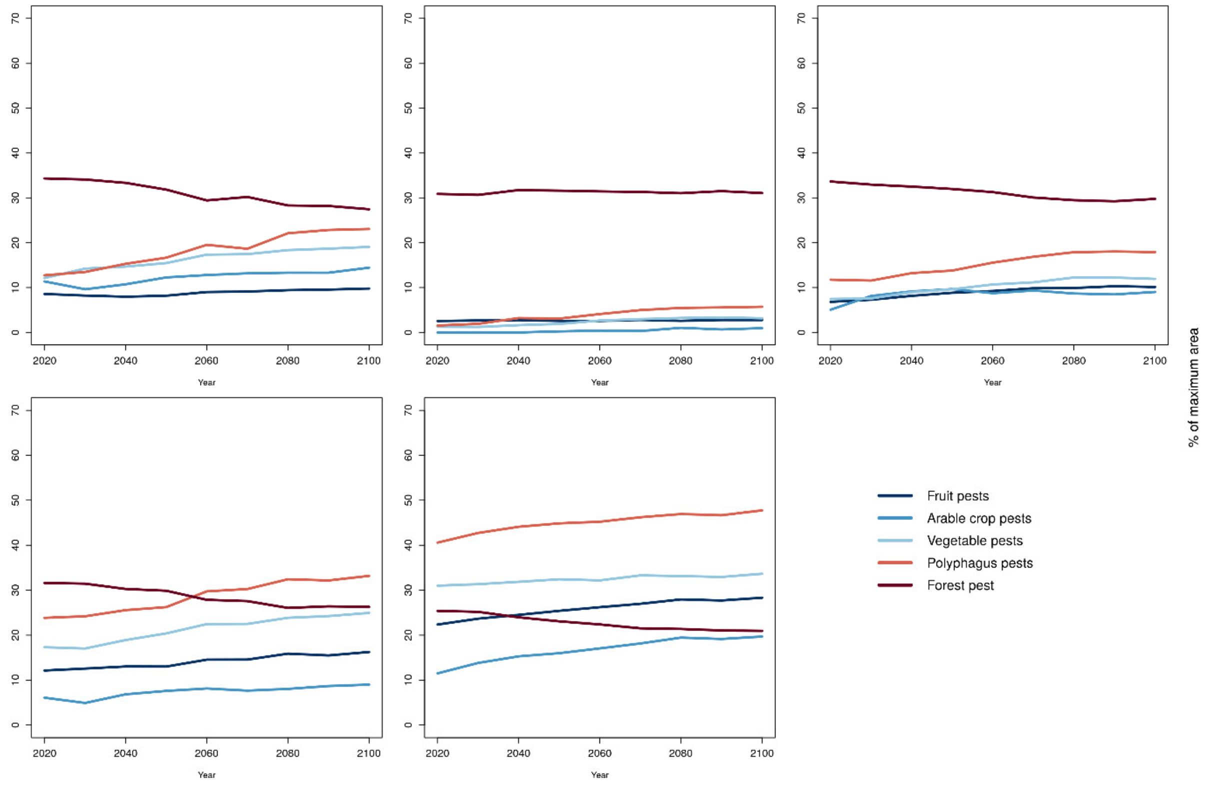

Supplementary Figure 10: Same as fig. S9 but under RCP4.5 scenario.

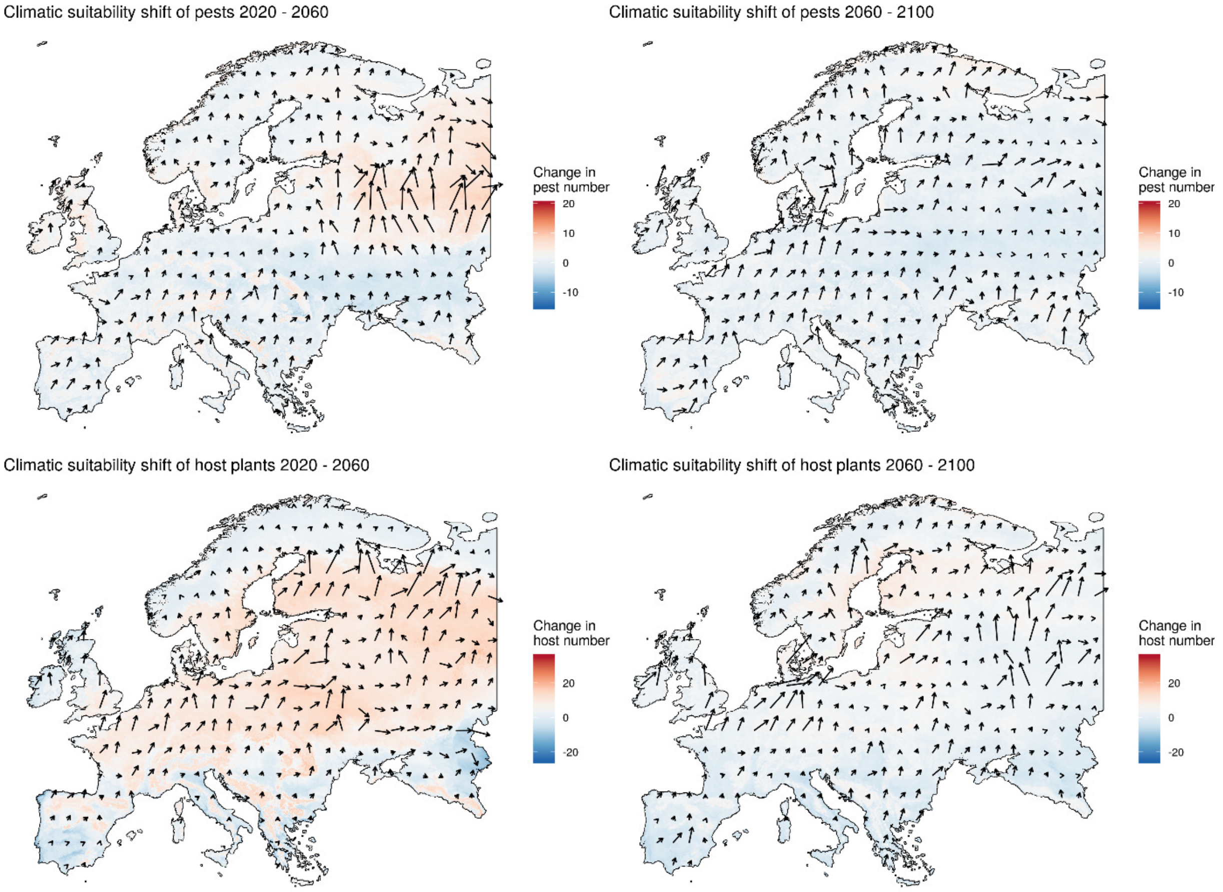

Supplementary Figure 11: Movement patterns of pests and host plants, same as figure 4 but for RCP4.5.

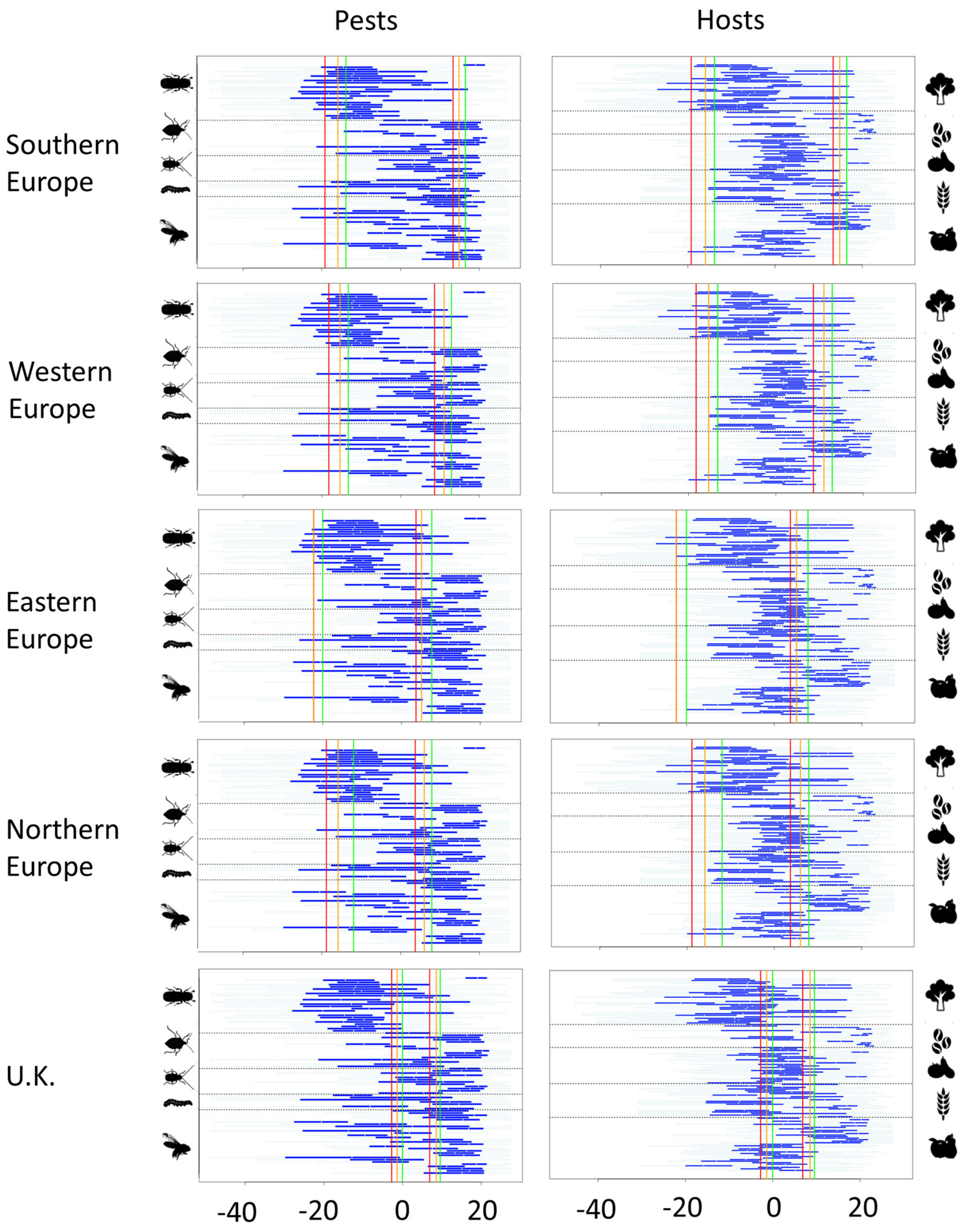

Supplementary Figure 12: Niche distribution of pests (left) and host plants (right) over Min Temp. Blue boxes show the niche of each pest. Vertical lines show the border of Min Temp conditions in Europe (red for 2020, orange for 2060 and green for 2100). Dashed horizontal lines are borders of pest categories. From bottom to top: fruit pests, arable crop pests, vegetable pests, polyphagous pests, forest pests.

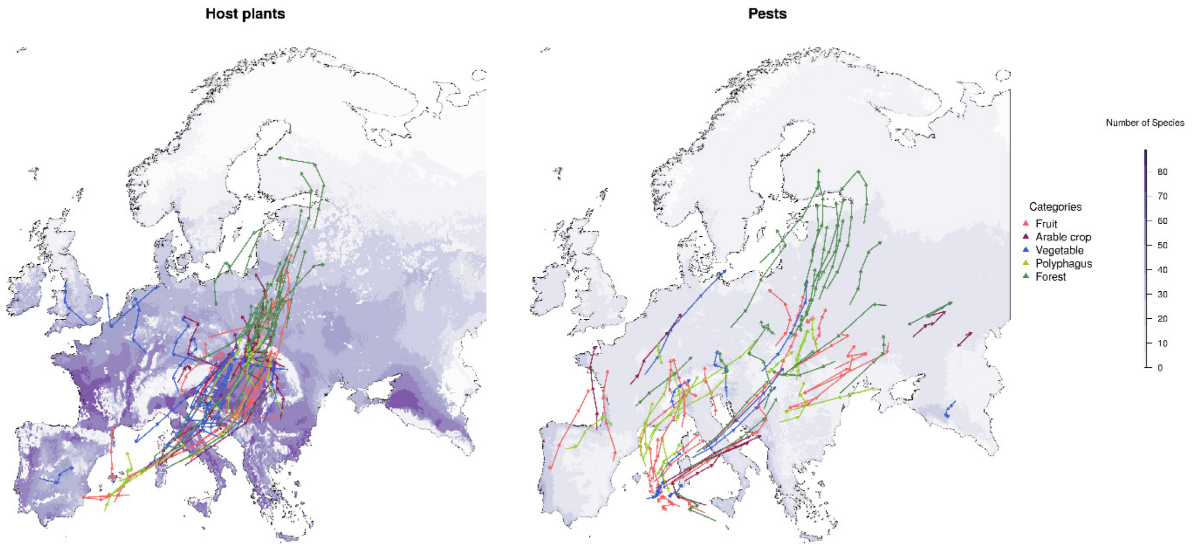

Supplementary Figure 13: Centroid shift between 2020 and 2100 in four time steps (2020 – 2040, 2040 – 2060, 2060 – 2080, 2080 – 2100) for the five different categories of host plants (left) and pests (right). Centroids were calculated on the individual distribution maps under the RCP8.5 scenario.

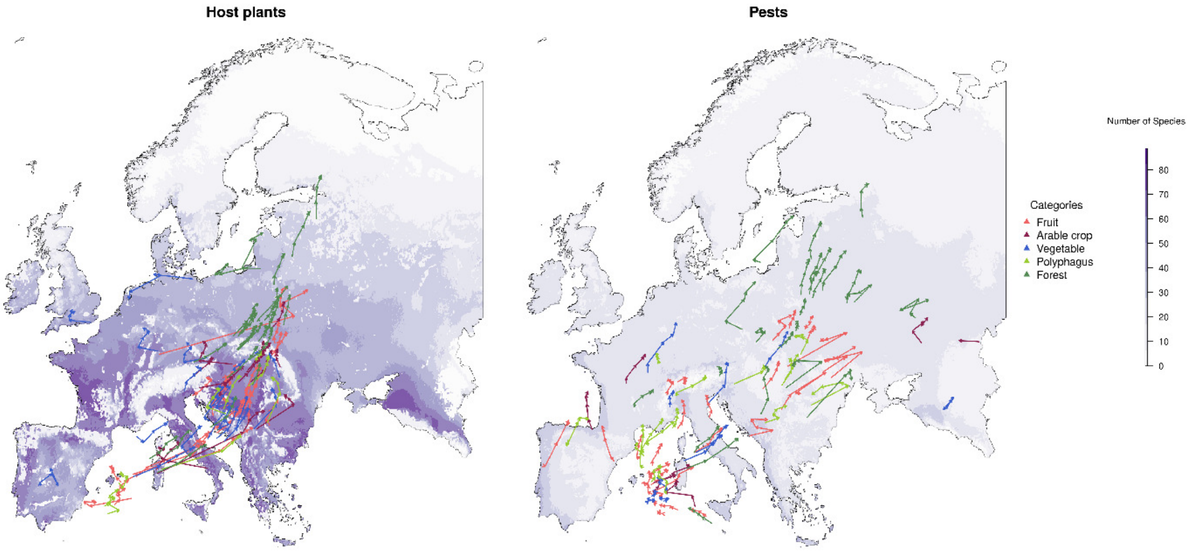

Supplementary Figure 14: Same as figure S12 but for RCP4.5.
