# Supplementary material for: Crop and forest pest metawebs shift towards increased linkage and suitability overlap under climate change
Source: Commun Biol. 2020 May 11;3:233. doi: 10.1038/s42003-020-0962-9 (PMC7214431; doi:10.1038/s42003-020-0962-9)
Supplement: Supplementary file 9 — Description of Additional Supplementary Files [file 42003_2020_962_MOESM9_ESM.pdf]

Description of Additional Supplementary Files

File Name: **Supplementary Data 1**
Description: List of pest species included in the study and variables used for the SDMs. Categories: 1 = Fruit pests, 2 = Arabale crop pests, 3 = Vegetable pests, 4 = Polyphagous pests, 5 = Forest pests

File Name: **Supplementary Data 2**
Description: List of host plant species included in the study and variables used for the SDMs. Categories: 1 = Fruit crops, 2 = Arabale crops, 3 = Vegetables, 4 = Other crops, 5 = Forest trees

File Name: **Supplementary Data 3**
Description: Pest model evaluation metrics: AUC scores and TSS values of the ensemble of each species and average of all GCMs and RCPs

File Name: **Supplementary Data 4**
Description: Host model evaluation metrics: AUC scores and TSS values of the ensemble of each species and average of all GCMs and RCPs.

File Name: **Supplementary Data 5**
Description: Area with suitable climatic habitat of all host plants under RCP8.5.

File Name: **Supplementary Data 6**
Description: Area with suitable climatic habitat of all host plants under RCP4.5.

File Name: **Supplementary Data 7**
Description: Pest species and description of their native and invasive range and the geographical origin of their occurrence records.
