# Supplementary material for: Crop and forest pest metawebs shift towards increased linkage and suitability overlap under climate change
Source: Commun Biol. 2020 May 11;3:233. doi: 10.1038/s42003-020-0962-9 (PMC7214431; doi:10.1038/s42003-020-0962-9)
Supplement: Supplementary file 10 — Reporting Summary [file 42003_2020_962_MOESM10_ESM.pdf]

nature research

Corresponding author(s): Marc Grünig

Last updated by author(s): Apr 9, 2020

Reporting Summary

Nature Research wishes to improve the reproducibility of the work that we publish. This form provides structure for consistency and transparency in reporting. For further information on Nature Research policies, see Authors & Referees and the Editorial Policy Checklist.

Statistics

For all statistical analyses, confirm that the following items are present in the figure legend, table legend, main text, or Methods section.

n/a	Confirmed	
☐	☒	The exact sample size (n) for each experimental group/condition, given as a discrete number and unit of measurement
☒	☐	A statement on whether measurements were taken from distinct samples or whether the same sample was measured repeatedly
☒	☐	The statistical test(s) used AND whether they are one- or two-sided<br>*Only common tests should be described solely by name; describe more complex techniques in the Methods section.*
☐	☒	A description of all covariates tested
☐	☒	A description of any assumptions or corrections, such as tests of normality and adjustment for multiple comparisons
☒	☐	A full description of the statistical parameters including central tendency (e.g. means) or other basic estimates (e.g. regression coefficient) AND variation (e.g. standard deviation) or associated estimates of uncertainty (e.g. confidence intervals)
☒	☐	For null hypothesis testing, the test statistic (e.g. F, t, r) with confidence intervals, effect sizes, degrees of freedom and P value noted<br>*Give P values as exact values whenever suitable.*
☒	☐	For Bayesian analysis, information on the choice of priors and Markov chain Monte Carlo settings
☒	☐	For hierarchical and complex designs, identification of the appropriate level for tests and full reporting of outcomes
☒	☐	Estimates of effect sizes (e.g. Cohen's d, Pearson's r), indicating how they were calculated

Our web collection on statistics for biologists contains articles on many of the points above.

Software and code

Policy information about availability of computer code

Data collection	EPPO PQR database (version 5.3.5) to get a list of potential hosts for each pest
Data analysis	R version 3.5.1 for all analysis. The R codes for the calculations of the results and analysis are available from the corresponding author. The code is also published under hhttps://doi.org/10.5281/zenodo.3746103

For manuscripts utilizing custom algorithms or software that are central to the research but not yet described in published literature, software must be made available to editors/reviewers. We strongly encourage code deposition in a community repository (e.g. GitHub). See the Nature Research guidelines for submitting code & software for further information.

Data

Policy information about availability of data

All manuscripts must include a data availability statement. This statement should provide the following information, where applicable:

- Accession codes, unique identifiers, or web links for publicly available datasets
- A list of figures that have associated raw data
- A description of any restrictions on data availability

The datasets of species occurrence records gathered during the current study is available from the corresponding author upon request.

Field-specific reporting

Please select the one below that is the best fit for your research. If you are not sure, read the appropriate sections before making your selection.

☐ Life sciences ☐ Behavioural & social sciences ☒ Ecological, evolutionary & environmental sciences

Ecological, evolutionary & environmental sciences study design

All studies must disclose on these points even when the disclosure is negative.

Study description	We use species distribution models to investigate the potential distribution of 89 insect pests and their host plants (126 crops and forest trees).
Research sample	89 insect pests and 126 crops and forest tree species.
Sampling strategy	We put together a list of pest species derived from the EPPO plant quarantine lists (Alert, A1, A2; www.eppo.int). We considered all crops for which distribution ranges are available from Earthstat (www.earthstat.org) and economically important forest tree species of Europe.
Data collection	We downloaded distribution ranges for crops as raster from Earthstat and for forest trees as shapefiles from EUFORGEN (www.euforgen.org) whenever available and presence records from GBIF (www.gbif.org) for all other species. We only included crops and forest trees listed as host plants for at least one of the pest species included in the EPPO plant quarantine lists (Alert, A1, A2; www.eppo.int). Vectors of plant pathogens were not considered. Occurrence records for pests were collected from various databases and from the published literature (see complete list of host plants and pests in Supplementary Tables 1 & 2). Data collection was done by Marc Gruenig
Timing and spatial scale	1.3.2017 - 1.9.2017
Data exclusions	To prevent from multiple records per cell and reduce sampling bias, we filtered the data with a minimum distance between each pair of records. With the remaining occurrence records the geographic extent of the species range is represented as reliably as possible (i.e. records in native and invasive range). To secure adequate SDM performance, we excluded species with fewer than 24 occurrence records. Models were considered to have a reliable performance with AUC scores > 0.7 and TSS values > 0.4. Models with AUC < 0.7 were not included.
Reproducibility	We used the set.seed function of the base package in R whenever subsampling was done.
Randomization	No experiments were carried out.
Blinding	No allocation of groups took place.

Did the study involve field work? ☐ Yes ☒ No

Field work, collection and transport

Field conditions	NA
Location	NA
Access and import/export	NA
Disturbance	NA

Reporting for specific materials, systems and methods

We require information from authors about some types of materials, experimental systems and methods used in many studies. Here, indicate whether each material, system or method listed is relevant to your study. If you are not sure if a list item applies to your research, read the appropriate section before selecting a response.

Materials & experimental systems

n/a	Involved in the study	
☒	☐	Antibodies
☒	☐	Eukaryotic cell lines
☒	☐	Palaeontology
☒	☐	Animals and other organisms
☒	☐	Human research participants
☒	☐	Clinical data

Methods

n/a	Involved in the study	
☒	☐	ChIP-seq
☒	☐	Flow cytometry
☒	☐	MRI-based neuroimaging
